# Supplementary material for: Do community-level factors play a role in HIV self-testing uptake, linkage to services and HIV-related outcomes? A mixed methods study of community-led HIV self-testing in rural Zimbabwe
Source: PLOS Glob Public Health. 2025 Apr 24;5(4):e0003196. doi: 10.1371/journal.pgph.0003196 (PMC12021296; doi:10.1371/journal.pgph.0003196)
Supplement: S2 Data — (PDF) [file pgph.0003196.s002.pdf]

Variable number	Question	Variable name	Response levels	Variable Format	Display format	Value label name	Variable label
1	Province	province	Text	String	%22s		Location
2	District name	districtname	Text	String	%10s		Location
3	Ward	ward	Text	String	%9s		Ward
4	ea	ea	Text	String	Integer		Enumeration Area
6	Name of headman unit	headmanunit_no	Text	String	%10.0g		Headman Unit
7	What is the study arm?	studyarm	CBD	String	%9s		Allocation
			CLD				
8	Individual ID	ptid	Alpha-numeric	String	%14s		IE09. Participant ID
29	ZIE6. Is this a household head or rep?	zie6	1 Household head	Numeric	%18.0g	HHREP	ZIE6. Is this a household head or rep?
			2 Household head rep				
			3 Not head/rep				
31	A3. Sex (Interviewer to indicate)	a3	1 Male	Numeric	%10.0g	RESPSEX	A3. Sex (Interviewer to indicate)
			2 Female				
43	HS01. Does your household have or own: Electricity	hs01_1	0 No	Numeric	%3.0f	YN	HS01. Electricity
			1 Yes				
44	HS01. Does your household have or own: Radio	hs01_2	0 No	Numeric	%3.0f	YN	HS01. Radio
			1 Yes				
45	HS01. Does your household have or own: Working Television	hs01_3	0 No	Numeric	%3.0f	YN	HS01. Working television
			1 Yes				
46	HS01. Does your household have or own: Mobile phone	hs01_4	0 No	Numeric	%3.0f	YN	HS01. Mobile phone
			1 Yes				
47	HS01. Does your household have or own: Non mobile phone	hs01_5	0 No	Numeric	%3.0f	YN	HS01. Non-mobile telephone (landline)
			1 Yes				
48	HS01. Does your household have or own: Refrigerator	hs01_6	0 No	Numeric	%3.0f	YN	HS01. Refrigerator
			1 Yes				
49	HS01. Does your household have or own: Bed with matress	hs01_7	0 No	Numeric	%3.0f	YN	HS01. Bed with mattress
			1 Yes				
50	HS01. Does your household have or own: Working car or truck	hs01_8	0 No	Numeric	%3.0f	YN	HS01. Working automobile or Car or Truck
			1 Yes				
51	HS01. Does your household have or own: Motorcycle	hs01_9	0 No	Numeric	%3.0f	YN	HS01. Motorcycle
			1 Yes				
52	HS01. Does your household have or own: Bicycle	hs01_10	0 No	Numeric	%3.0f	YN	HS01. Bicycle
			1 Yes				
53	HS01. Does your household have or own: Table and chair	hs01_11	0 No	Numeric	%3.0f	YN	HS01. Table and chair
			1 Yes				
55	HS02.Does any member of this household own: Mobile phone	hs02a	0 No	Numeric	%18.0g	YES_NO_DTA	HS02a, Mobile phone
			1 Yes				
			9 Declined to answer				
56	HS02.Does any member of this household own: Car or Truck	hs02b	0 No	Numeric	%18.0g	YES_NO_DTA	HS02b. Working automobile (Car or Truck)
			1 Yes				
			9 Declined to answer				
57	HS02.Does any member of this household own: Bicycle	hs02c	0 No	Numeric	%18.0g	YES_NO_DTA	HS02c. Bicycle
			1 Yes				
			9 Declined to answer				
58	HS02.Does any member of this household own: Working motorcycle or motor scooter	hs02d	0 No	Numeric	%18.0g	YES_NO_DTA	HS02d. Working motorcycle or motor scooter
			1 Yes				
			9 Declined to answer				
59	HS02.Does any member of this household own: An animal-drawn cart	hs02e	0 No	Numeric	%18.0g	YES_NO_DTA	HS02e. An animal-drawn cart
			1 Yes				
			9 Declined to answer				
60	HS04. Have you or anyone in your household raised or owned cattle during the pas	hs04	0 No	Numeric	%18.0g	YES_NO_DTA	HS04. Have you or anyone in your household raised or owned cattle during the pas
			1 Yes				
			9 Declined to answer				
71	A05. What was the highest level of education that you have completed?	a05	1 No formal schooling	Numeric	%30.0g	HOHEDU1	A05. What was the highest level of education that you have completed?
			2 Primary incomplete or complete				
			3 Some secondary education				
			4 O-levels complete				
			5 A-levels complete				
			6 College or higher				
74	ZA12. What is your religion?	za12	1 Roman Catholic	Numeric	%28.0g	RELIGION	ZA12. What is your religion?
			2 Anglican				
			3 Lutheran				
			4 Methodist				
			5 Baptist				
			6 Presbyterian				
			7 Apostolic				
			8 Pentecostal				
			9 Moslem				
			10 African traditional religion				
			11 Other				
			12 No religion				
76	ZA10. What is your current occupation?	za10	1 Student	Numeric	%18.0g	OCCUPATION	ZA10. What is your current occupation?
			2 Subsistence farmer				
			3 Self-employed				
			4 Formal employment				
77	A08. In a month, how much do you earn for wage/salary and commission before taxe	a08	Numeric	Numeric	Integer		A08. In a month, how much do you earn for wage/salary and commission before taxe
82	A12. How do you rate your general health	a12	1 Very good	Numeric	%22.0g	HEALTH	A12. How do you rate your general health
			2 Good				
			3 Fair				
			4 Poor				
			9 I don't wish to answer				
85	A16. What is your current marital status?	a16	1 Married or living as married	Numeric	%28.0g	MSTATUS	A16. What is your current marital status?
			2 Never married				
			3 Widowed/separated/divorced				
			9 Decline to answer				
86	ZA15. Is this your first marriage?	za15	1 Yes, first marriage	Numeric	%27.0g	FIRST_MARRIAGE	ZA15. Is this your first marriage?
			2 No, remarried after divorce				
			3 No, remarried after widowed				
90	A19. Have you ever lost a spouse due to death?	a19	0 No	Numeric	%10.0g	YES_NO	A19. Have you ever lost a spouse due to death?
			1 Yes				
92	AX1. Who usually makes decisions about health care for yourself?	ax1	1 You	Numeric	%37.0g	DECISION	AX1. Who usually makes decisions about health care for yourself?
			2 Your partner				
			3 You or your partner or spouse jointly				
			4 Parent/ Guardian				
			5 Someone else				
93	AX2. Who usually makes decisions about major household purchases?	ax2	1 You	Numeric	%37.0g	DECISION	AX2. Who usually makes decisions about major household purchases?
			2 Your partner				
			3 You or your partner or spouse jointly				
			4 Parent/ Guardian				

			5 Someone else				
94	AX3. Who usually makes decisions about visits to your family or relatives?	ax3	1 You	Numeric	%37.0g	DECISION	AX3. Who usually makes decisions about visits to your family or relatives?
			2 Your partner				
			3 You or your partner or spouse jointly				
			4 Parent/ Guardian				
			5 Someone else				
139	CM04. People in this village are willing to help their neighbours	cm04	1 Strongly agree	Numeric	%26.0g	COHESION	CM04. People in this village are willing to help their neighbours
			2 Somewhat agree				
			3 Neither agree nor disagree				
			4 Somewhat disagree				
			5 Strongly disagree				
140	CM05. This is a close knit community	cm05	1 Strongly agree	Numeric	%26.0g	COHESION	CM05. This is a close knit community
			2 Somewhat agree				
			3 Neither agree nor disagree				
			4 Somewhat disagree				
			5 Strongly disagree				
141	CM06. People in this village can be trusted	cm06	1 Strongly agree	Numeric	%26.0g	COHESION	CM06. People in this village can be trusted
			2 Somewhat agree				
			3 Neither agree nor disagree				
			4 Somewhat disagree				
			5 Strongly disagree				
142	CM07. People in this village generally get along well with each other	cm07	1 Strongly agree	Numeric	%26.0g	COHESION	CM07. People in this village generally get along well with each other
			2 Somewhat agree				
			3 Neither agree nor disagree				
			4 Somewhat disagree				
			5 Strongly disagree				
143	CM08. People in this village share the same values	cm08	1 Strongly agree	Numeric	%26.0g	COHESION	CM08. People in this village share the same values
			2 Somewhat agree				
			3 Neither agree nor disagree				
			4 Somewhat disagree				
			5 Strongly disagree				
144	CM09. People in this village look out for each other	cm09	1 Strongly agree	Numeric	%26.0g	COHESION	CM09. People in this village look out for each other
			2 Somewhat agree				
			3 Neither agree nor disagree				
			4 Somewhat disagree				
			5 Strongly disagree				
145	CM10. People in your village are concerned about HIV	cm10	1 Strongly agree	Numeric	%26.0g	COHESION	CM10. People in your village are concerned about HIV
			2 Somewhat agree				
			3 Neither agree nor disagree				
			4 Somewhat disagree				
			5 Strongly disagree				
146	CM11. People in your village consider HIV/AIDS an important issue	cm11	1 Strongly agree	Numeric	%26.0g	COHESION	CM11. People in your village consider HIV/AIDS an important issue
			2 Somewhat agree				
			3 Neither agree nor disagree				
			4 Somewhat disagree				
			5 Strongly disagree				
147	CM12. People in your village talk openly about HIV	cm12	1 Strongly agree	Numeric	%26.0g	COHESION	CM12. People in your village talk openly about HIV
			2 Somewhat agree				
			3 Neither agree nor disagree				
			4 Somewhat disagree				
			5 Strongly disagree				
148	CM13. People in your village believe that HIV impacts the community	cm13	1 Strongly agree	Numeric	%26.0g	COHESION	CM13. People in your village believe that HIV impacts the community
			2 Somewhat agree				
			3 Neither agree nor disagree				
			4 Somewhat disagree				
			5 Strongly disagree				
149	CM14. People in your village talk about HIV/AIDS at community meetings	cm14	1 Strongly agree	Numeric	%26.0g	COHESION	CM14. People in your village talk about HIV/AIDS at community meetings
			2 Somewhat agree				
			3 Neither agree nor disagree				
			4 Somewhat disagree				
			5 Strongly disagree				
150	CM15. People in your village work together to prevent HIV from spreading	cm15	1 Strongly agree	Numeric	%26.0g	COHESION	CM15. People in your village work together to prevent HIV from spreading
			2 Somewhat agree				
			3 Neither agree nor disagree				
			4 Somewhat disagree				
			5 Strongly disagree				
151	CM16. People in your village work together to reduce the effects of HIV	cm16	1 Strongly agree	Numeric	%26.0g	COHESION	CM16. People in your village work together to reduce the effects of HIV
			2 Somewhat agree				
			3 Neither agree nor disagree				
			4 Somewhat disagree				
			5 Strongly disagree				
152	CM17. People in your village believe they can change the course of the HIV/AIDS	cm17	1 Strongly agree	Numeric	%26.0g	COHESION	CM17. People in your village believe they can change the course of the HIV/AIDS
			2 Somewhat agree				
			3 Neither agree nor disagree				
			4 Somewhat disagree				
			5 Strongly disagree				
153	CM18. People in your village exchange information about HIV/AIDS	cm18	1 Strongly agree	Numeric	%26.0g	COHESION	CM18. People in your village exchange information about HIV/AIDS
			2 Somewhat agree				
			3 Neither agree nor disagree				
			4 Somewhat disagree				
			5 Strongly disagree				
154	CM19. People in your village take HIV/AIDS seriously	cm19	1 Strongly agree	Numeric	%26.0g	COHESION	CM19. People in your village take HIV/AIDS seriously
			2 Somewhat agree				
			3 Neither agree nor disagree				
			4 Somewhat disagree				
			5 Strongly disagree				
155	CM20. People work together to solve problems in the village	cm20	1 Strongly agree	Numeric	%26.0g	COHESION	CM20. People work together to solve problems in the village
			2 Somewhat agree				
			3 Neither agree nor disagree				
			4 Somewhat disagree				
			5 Strongly disagree				
156	CM21. People in your village talk to each other about how to solve village probl	cm21	1 Strongly agree	Numeric	%26.0g	COHESION	CM21. People in your village talk to each other about how to solve village probl
			2 Somewhat agree				
			3 Neither agree nor disagree				
			4 Somewhat disagree				
			5 Strongly disagree				
157	CM22. People in your village enjoy discussing different ways to solve village pr	cm22	1 Strongly agree	Numeric	%26.0g	COHESION	CM22. People in your village enjoy discussing different ways to solve village pr
			2 Somewhat agree				
			3 Neither agree nor disagree				
			4 Somewhat disagree				
			5 Strongly disagree				
158	CM23. People in your village are open to hearing different views about community	cm23	1 Strongly agree	Numeric	%26.0g	COHESION	CM23. People in your village are open to hearing different views about community
			2 Somewhat agree				
			3 Neither agree nor disagree				
			4 Somewhat disagree				
			5 Strongly disagree				

159	CM24. People in your village volunteer to help solve village problems	cm24	1 Strongly agree	Numeric	%26.0g	COHESION	CM24. People in your village volunteer to help solve village problems
			2 Somewhat agree				
			3 Neither agree nor disagree				
			4 Somewhat disagree				
			5 Strongly disagree				
160	CM25. People in your village think about why there are problems so they can addr	cm25	1 Strongly agree	Numeric	%26.0g	COHESION	CM25. People in your village think about why there are problems so they can addr
			2 Somewhat agree				
			3 Neither agree nor disagree				
			4 Somewhat disagree				
			5 Strongly disagree				
161	CM26. There is a lot of cooperation between groups in the village	cm26	1 Strongly agree	Numeric	%26.0g	COHESION	CM26. There is a lot of cooperation between groups in the village
			2 Somewhat agree				
			3 Neither agree nor disagree				
			4 Somewhat disagree				
			5 Strongly disagree				
162	CM27. People in this village not only talk about problems but they also try to s	cm27	1 Strongly agree	Numeric	%26.0g	COHESION	CM27. People in this village not only talk about problems but they also try to s
			2 Somewhat agree				
			3 Neither agree nor disagree				
			4 Somewhat disagree				
			5 Strongly disagree				
163	CM28. If your community fails to resolve a community problem, they will try anot	cm28	1 Strongly agree	Numeric	%26.0g	COHESION	CM28. If your community fails to resolve a community problem, they will try anot
			2 Somewhat agree				
			3 Neither agree nor disagree				
			4 Somewhat disagree				
			5 Strongly disagree				
164	CM29. If your community fails to resolve a community problem, they will learn fr	cm29	1 Strongly agree	Numeric	%26.0g	COHESION	CM29. If your community fails to resolve a community problem, they will learn fr
			2 Somewhat agree				
			3 Neither agree nor disagree				
			4 Somewhat disagree				
			5 Strongly disagree				
165	CM30. If leaders in the village fail to resolve a village problem, the villagers	cm30	1 Strongly agree	Numeric	%26.0g	COHESION	CM30. If leaders in the village fail to resolve a village problem, the villagers
			2 Somewhat agree				
			3 Neither agree nor disagree				
			4 Somewhat disagree				
			5 Strongly disagree				
166	B01 - Have you ever been tested for HIV? This includes all types of HIV tests in	b01	0 No	Numeric	%10.0g	YES_NO	B01 - Have you ever been tested for HIV? This includes all types of HIV tests in
			1 Yes				
186	B05 - Do you know any facilities offering HIV testing and counselling to people	b05	0 No	Numeric	%10.0g	YES_NO	B05 - Do you know any facilities offering HIV testing and counselling to people
			1 Yes				
187	B06 - If you wanted to go, how easy or difficult would it be for you to go the h	b06	1 Very easy	Numeric	%18.0g	EASYDIFF	B06 - If you wanted to go, how easy or difficult would it be for you to go the h
			2 Somewhat easy				
			3 Somewhat difficult				
			4 Very difficult				
			8 Don't know				
			9 Declined to answer				
188	B07a - Have you ever had an HIV test offered to you, including a self-test? The	b07a	0 No	Numeric	%10.0g	YES_NO	B07a - Have you ever had an HIV test offered to you, including a self-test? The
			1 Yes				
196	B14 - Have you ever used a self-test to test for HIV?	b14	0 No	Numeric	%10.0g	YES_NO	B14 - Have you ever used a self-test to test for HIV?
			1 Yes				
198	B15 - Within the past 12 months, have you used a self-test to test for HIV?	b15	0 No	Numeric	byte	YES_NO	B15 - Within the past 12 months, have you used a self-test to test for HIV?
			1 Yes				
255	ZB27 - What was the result of your self-test?	zb27	1 Positive	Numeric	byte	HIVRESULT	ZB27 - What was the result of your self-test?
			2 Negative				
279	ZB28 - Did you go to have an HIV test in order to confirm the results you obtain	zb28	0 No	Numeric	byte	YES_NO	ZB28 - Did you go to have an HIV test in order to confirm the results you obtain
			1 Yes				
283	ZB31 - What was the result of the confirmatory testing?	zb31	1 Positive	Numeric	%17.0g	HIVRESULT2	ZB31 - What was the result of the confirmatory testing?
			2 Negative				
			3 Prefer not to say				
288	ZB34. After you obtained an HIV negative result, did you seek medical male circi	zb34	1 Yes, and I was circumcised	Numeric	%30.0g	LINKVMMC	ZB34. After you obtained an HIV negative result, did you seek medical male circi
			2 Yes, but I was not circumcised				
			3 No				
301	ZB35b - After self-testing did you seek to protect yourself from HIV through PrEP	zb35b	1 Yes, and I was started on PrEP	Numeric	%58.0g	LINKPREP	ZB35b - After self-testing did you seek to protect yourself from HIV through PrE
			2 Yes but I was not given or started on PrEP				
			3 No, I did not need PrEP because I was already HIV positive				
			4 No, I do not consider myself at risk of getting HIV				
			5 No, I did not know about PrEP				
			6 No, I did not seek PrEP for another reason				
304	ZB35e - Did you start PrEP before or after the the self-testing program came to	zb35e	1 Before the self-testing program came to the community	Numeric	%53.0g	PREPBEFOREAFTER	ZB35e - Did you start PrEP before or after the the self-testing program came to
			2 After the self-testing program came to the community				
309	C03_1 - Was this test a self-test?	c03_1	0 No	Numeric	%10.0g	YES_NO	C03_1 - Was this test a self-test?
			1 Yes				
341	C24_1 - You don't have to tell me if you dont want to, but what were the results?	c24_1	1 Positive	Numeric	%20.0g	KNOWRESULTS	C24_1 - You don't have to tell me if you dont want to, but what were the results
			2 Negative				
			3 Indeterminate				
			4 Prefer not to reveal				
383	C32_1 - After this HIV test, did you receive a test confirming your HIV diagnosi	c32_1	0 No	Numeric	%10.0g	YES_NO	C32_1 - After this HIV test, did you receive a test confirming your HIV diagnosi
			1 Yes				
384	C33_1 - What care did you receive?	c33_1	1 Only follow-up test to confirm the results of the test	Numeric	%73.0g	AFTERDET_X	C33_1 - What care did you receive?
			2 Started treatment for HIV (antiretroviral treatment) for the first time				
			3 Restarted treatment for HIV (antiretroviral treatment) that I had stopped				
			4 Other care (not including either confirmatory test or ART)				
			9 Decline to answer				
401	C35_1 - [Asked of men only:] Did you go for VMMC (voluntary medical male circumc	c35_1	0 No	Numeric	%10.0g	YES_NO	C35_1 - [Asked of men only:] Did you go for VMMC (voluntary medical male circumc
			1 Yes				
406	C03_2 - Was this test a self-test?	c03_2	0 No	Numeric	%10.0g	YES_NO	C03_2 - Was this test a self-test?
			1 Yes				
466	C32_2 - After this HIV test, did you receive a test confirming your HIV diagnosi	c32_2	0 No	Numeric	%10.0g	YES_NO	C32_2 - After this HIV test, did you receive a test confirming your HIV diagnosi
			1 Yes				
467	C33_2 - What care did you receive?	c33_2	1 Only follow-up test to confirm the results of the test	Numeric	%73.0g	AFTERDET_X	C33_2 - What care did you receive?
			2 Started treatment for HIV (antiretroviral treatment) for the first time				
			3 Restarted treatment for HIV (antiretroviral treatment) that I had stopped				
			4 Other care (not including either confirmatory test or ART)				
			9 Decline to answer				

489	C03 3 - Was this test a self-test?	c03 3	0 No	Numeric	byte	YES NO	C03 3 - Was this test a self-test?
			1 Yes				
549	C32_3 - After this HIV test, did you receive a test confirming your HIV diagnosi	c32_3	0 No	Numeric	byte	YES_NO	C32_3 - After this HIV test, did you receive a test confirming your HIV diagnosi
		c32_3	1 Yes	Numeric			
550	C33_3 - What care did you receive?	c33_3	1 Only follow-up test to confirm the results of the test	Numeric	byte	AFTERDET_X	C33_3 - What care did you receive?
			2 Started treatment for HIV (antiretroviral treatment) for the first time				
			3 Restarted treatment for HIV (antiretroviral treatment) that I had stopped				
			4 Other care (not including either confirmatory test or ART)				
			9 Decline to answer				
567	C35_3 - [Asked of men only:] Did you go for VMMC (voluntary medical male circumc	c35_3	0 No	Numeric	byte	YES_NO	C35_3 - [Asked of men only:] Did you go for VMMC (voluntary medical male circumc
			1 Yes				
571	F01. Have you ever had a positive HIV test result?	f01	0 No	Numeric	byte	YES_NO_DTA	F01. Have you ever had a positive HIV test result?
			1 Yes				
			9 Declined to answer				
572	F02. Did you obtain this result from a self-test?	f02	0 No	Numeric	byte	YES_NO_DTA	F02. Did you obtain this result from a self-test?
			1 Yes				
			9 Declined to answer				
573	F03. Did you go to a health facilty to confirm the positive self-test result?	f03	0 No	Numeric	byte	YES_NO_DTA	F03. Did you go to a health facilty to confirm the positive self-test result?
			1 Yes				
			9 Declined to answer				
596	G01. Do you have a steady partner?	g01	0 No	Numeric	byte	YES_NO_DTA	G01. Do you have a steady partner?
			1 Yes				
			9 Declined to answer				
597	G02. How many steady partners have you had sex with in the last 3 months? Enter	g02	Number	Numeric	byte		G02. How many steady partners have you had sex with in the last 3 months? Enter
598	G3. In the past 3 months has there been an occasion when you did not use condoms	g3	1 Condoms every time	Numeric	byte	CONDOM_USE	G3. In the past 3 months has there been an occasion when you did not use condoms
			2 Condoms some of the time				
			3 Condoms never used				
			9 Don't want to answer				
599	G03. In the past 3 months has there been an occasion when you did not use condom	g03_01	1 Condoms every time	Numeric	byte	CONDOM_USE	G03. In the past 3 months has there been an occasion when you did not use condom
			2 Condoms some of the time				
			3 Condoms never used				
			9 Don't want to answer				
600	G03. In the past 3 months has there been an occasion when you did not use condom	g03_02	1 Condoms every time	Numeric	byte	CONDOM_USE	
			2 Condoms some of the time				
			3 Condoms never used				
			9 Don't want to answer				
601	G03. In the past 3 months has there been an occasion when you did not use condom	g03_03	1 Condoms every time	Numeric	%24.0g	CONDOM_USE	
			2 Condoms some of the time				
			3 Condoms never used				
			9 Don't want to answer				
602	G03. In the past 3 months has there been an occasion when you did not use condom	g03_04	1 Condoms every time	Numeric	byte	CONDOM_USE	
			2 Condoms some of the time				
			3 Condoms never used				
			9 Don't want to answer				
603	G03. In the past 3 months has there been an occasion when you did not use condom	g03_05	1 Condoms every time	Numeric	byte	CONDOM_USE	
			2 Condoms some of the time				
			3 Condoms never used				
			9 Don't want to answer				
604	G03. In the past 3 months has there been an occasion when you did not use condom	g03_06	1 Condoms every time	Numeric	byte	CONDOM_USE	
			2 Condoms some of the time				
			3 Condoms never used				
			9 Don't want to answer				
605	G04. Apart from your steady partner(s), have you had sex with anyone else in the	g04	0 No	Numeric	byte	YES_NO_DTA	G04. Apart from your steady partner(s), have you had sex with anyone else in the
			1 Yes				
			9 Declined to answer				
606	G05. With how many people apart from your steady partner have you had sex withou	g05	Number	Numeric	byte		G05. With how many people apart from your steady partner have you had sex withou
620	H03. People sometimes talk badly about people living with or thought to be livin	h03	1 Strongly Agree	Numeric	byte	SITGCHILD	H03. People sometimes talk badly about people living with or thought to be livin
			2 Agree				
			3 Unsure				
			4 Disagree				
			5 Strongly disagree				
622	H05. People living with or thought to be living with HIV lose respect or standin	h05	1 Strongly Agree	Numeric	byte	SITGCHILD	H05. People living with or thought to be living with HIV lose respect or standin
			2 Agree				
			3 Unsure				
			4 Disagree				
			5 Strongly disagree				
623	H06. People living with or thought to be living with HIV are verbally insulted,	h06	1 Strongly Agree	Numeric	byte	SITGCHILD	H06. People living with or thought to be living with HIV are verbally insulted,
			2 Agree				
			3 Unsure				
			4 Disagree				
			5 Strongly disagree				
624	H07. People living with or thought to be living with HIV are sometimes physicall	h07	1 Strongly Agree	Numeric	byte	SITGCHILD	H07. People living with or thought to be living with HIV are sometimes physicall
			2 Agree				
			3 Unsure				
			4 Disagree				
			5 Strongly disagree				
625	H08. I would be ashamed if someone in my family had HIV	h08	1 Strongly Agree	Numeric	byte	SITGCHILD	H08. I would be ashamed if someone in my family had HIV
			2 Agree				
			3 Unsure				
			4 Disagree				
			5 Strongly disagree				
626	H09. I would not like to sit close to someone living with HIV,for example on pub	h09	1 Strongly Agree	Numeric	byte	SITGCHILD	H09. I would not like to sit close to someone living with HIV,for example on pub
			2 Agree				
			3 Unsure				
			4 Disagree				
			5 Strongly disagree				
627	H10. I fear that I could contract HIV if I come into contact with the saliva of	h10	1 Strongly Agree	Numeric	byte	SITGCHILD	H10. I fear that I could contract HIV if I come into contact with the saliva of
			2 Agree				
			3 Unsure				
			4 Disagree				
			5 Strongly disagree				
628	H11. People sometimes disclose that other people are HIV positive without their	h11	1 Strongly Agree	Numeric	byte	SITGCHILD	H11. People sometimes disclose that other people are HIV positive without their
			2 Agree				
			3 Unsure				
			4 Disagree				
			5 Strongly disagree				
629	H12. Health workers sometimes disclose that other people are HIV positive withou	h12	1 Strongly Agree	Numeric	byte	SITGCHILD	H12. Health workers sometimes disclose that other people are HIV positive withou
			2 Agree				
			3 Unsure				

			4 Disagree				
			5 Strongly disagree				
630	H13. People living with HIV who are taking ARVs are treated better by others tha	h13	1 Strongly Agree	Numeric	byte	SITGCHILD	H13. People living with HIV who are taking ARVs are treated better by others tha
			2 Agree				
			3 Unsure				
			4 Disagree				
			5 Strongly disagree				
633	P03. I would feel safe having sex with someone who is HIV-positive as long as th	p03	1 Strongly Agree	Numeric	byte	KNOWLEDGE	P03. I would feel safe having sex with someone who is HIV-positive as long as th
			2 Agree				
			3 Unsure				
			4 Disagree				
			5 Strongly disagree				
			9 Decline to answer				
634	P04. I am less worried about HIV infection than I used to be	p04	1 Strongly Agree	Numeric	byte	KNOWLEDGE	P04. I am less worried about HIV infection than I used to be
			2 Agree				
			3 Unsure				
			4 Disagree				
			5 Strongly disagree				
			9 Decline to answer				
635	P05. HIV treatment makes me less anxious about having unprotected sex	p05	1 Strongly Agree	Numeric	byte	KNOWLEDGE	P05. HIV treatment makes me less anxious about having unprotected sex
			2 Agree				
			3 Unsure				
			4 Disagree				
			5 Strongly disagree				
			9 Decline to answer				
639	P09. HIV treatment can help prevent a person with HIV from infecting a partner	p09	1 Strongly Agree	Numeric	byte	KNOWLEDGE	P09. HIV treatment can help prevent a person with HIV from infecting a partner
			2 Agree				
			3 Unsure				
			4 Disagree				
			5 Strongly disagree				
			9 Decline to answer				
666		ppt_dob		Date	Integer		PPT_DOB
667	Age of participant	respageyrs		Numeric	Integer		Age of participant
