# Supplementary material for: Do community-level factors play a role in HIV self-testing uptake, linkage to services and HIV-related outcomes? A mixed methods study of community-led HIV self-testing in rural Zimbabwe
Source: PLOS Glob Public Health. 2025 Apr 24;5(4):e0003196. doi: 10.1371/journal.pgph.0003196 (PMC12021296; doi:10.1371/journal.pgph.0003196)
Supplement: Attachment — Submitted filename: Response to reviewers.docx [file pgph.0003196.s004.docx]

09 February 2025

The Editor

PLOS Global Health

Dear Editor,

Re: Submission of revised manuscript to PLOS Global Health

Thank you for reviewing our manuscript titled “What role do community-level factors play in HIV self-testing uptake, linkage to services and HIV-related outcomes? A mixed methods study of community-led HIV self-testing programmes in rural Zimbabwe”. We are submitting our revised manuscript following receipt of reviewer comments. Please find below responses to each of the comments raised by Reviewer 1.

Reviewer #1:

1. The manuscript provides findings that are very relevant to the current programmatic gaps and provides insights to improve community testing specially for men who are still lagging behind in the first 95. I however suggest that the author clearly defines who the community is in the context of this paper, and the characteristics of the community described for purposes of evaluating the value added to the body of knowledge on the subject matter.

*Thank you for your positive feedback on our manuscript. In our context, the community refers to the general, adult population residing in adjacent villages that are headed by a local leader known as a headman. These include farming and mining communities served by a district hospital and/or rural health centre. We have now made clarifications in lines 157-159 (Methods section, under “Setting”) and 352-355 (Results section, under “Participant and community characteristics”).*

2. The author should also define what cohesion means in the context of the manuscript since it is one of the variables used in the analysis.

*Thank you. We used a validated 6-item measure of community cohesion and considered cohesion, in our context, as referring to individuals’ perceptions of the extent to which their community is closely knit, shares the same values, trusts each other, and members are willing to help each other. We have now included this definition in lines 234-236 (Methods section, under “Population-based survey”).*

3. It would be good to have the author explain if these findings are still relevant to the current Zimbabwe context noting that the self - testing strategy is now scaled up beyond the setting described in this paper.

*Thank you. We agree that self-testing is now scaled up in Zimbabwe. As Zimbabwe and similar countries approach HIV testing targets, there is evidence that some groups have remained behind, hence innovative testing models that effectively reach priority groups are still required, hence the findings are still very relevant. We have revised this in lines 83-88 (Introduction section).*

4. It will be important to get clarification from the author about the gap that self testing will address in the current community described in the manuscript, is it a gap filler, is it a strategy for the hard to reach or it is another prevention option for the populations in need.

*Thank you. We believe that to attain HIV testing and treatment targets, there is not a single intervention that will address all challenges and provide all the required solutions. Our study demonstrates community-led HIV self-testing as a feasible and acceptable model that better identifies those at most risk of undiagnosed HIV. In lines 506-535 in the Discussion we address the fact that there were other outcomes that were not improved by the intervention, showing that it is a tool that is important for targeting priority groups that can be included in a mixed bag of interventions that have public health impact.*

5. The author should explain how this self testing strategy will become part of the policy for future scale up and how sustainability can be achieved through embedment in policy.

*Thank you. It is essential to embed community-led HIVST into existing health frameworks and national HIV/AIDS strategies, to ensure that this strategy becomes part of policy and is scaled up in future. This can be achieved by stakeholder engagement including communities, researchers, technical partners and Ministries of Health. We have made mention of this in lines 588-592 (Discussion section).*

Finally, we have made additional, minor changes as follows: (i) correcting typographical errors and grammar, (ii) ensuring the use of consistent terminology and spellings (e.g., British spellings), (iii) improving readability, (iv) adding column headings in tables 5-7 (v) minor changes to the title of the paper and (vi) addition of one reference. All changes are tracked in the revised manuscript, for your review.

We hope that we have addressed these comments satisfactorily and look forward to hearing from you.

Yours faithfully,

Ms Mary K Tumushime

(Corresponding author)
